# Comparable seasonal pattern for COVID-19 and flu-like illnesses

**DOI:** 10.1016/j.onehlt.2021.100277

**Published:** 2021-06-08

**Authors:** Martijn J. Hoogeveen, Ellen K. Hoogeveen

**Affiliations:** a Department Technical Sciences & Environment, Open University, the Netherlands; b Department of Internal Medicine, Jeroen Bosch Hospital, Den Bosch, the Netherlands

**Keywords:** COVID-19 incidence, Seasonality, Influenza-like illnesses, Respiratory viruses

## Abstract

**Background:**

During the first wave of COVID-19 it was hypothesized that COVID-19 is subject to multi-wave seasonality, similar to Influenza-Like Illnesses since time immemorial. One year into the pandemic, we aimed to test the seasonality hypothesis for COVID-19.

**Methods:**

We calculated the average annual time-series for Influenza-Like Illnesses based on incidence data from 2016 till 2019 in the Netherlands, and compared these with two COVID-19 time-series during 2020/2021 for the Netherlands. We plotted the time-series on a standardized logarithmic infection scale. Finally, we calculated correlation coefficients and used univariate regression analysis to estimate the strength of the association between the time-series of COVID-19 and Influenza-Like Illnesses.

**Results:**

The time-series for COVID-19 and Influenza-Like Illnesses were strongly and highly significantly correlated. The COVID-19 peaks were all during flu season, and lows were all in the opposing period. Finally, COVID-19 meets the multi-wave characteristics of earlier flu-like pandemics, namely a short first wave at the tail-end of a flu season, and a longer and more intense second wave during the subsequent flu season.

**Conclusions:**

We conclude that seasonal patterns of COVID-19 incidence and Influenza-Like Illnesses incidence are highly similar, in a country in the temperate climate zone, such as the Netherlands. Further, the COVID-19 pandemic satisfies the criteria of earlier respiratory pandemics, namely a first wave that is short-lived at the tail-end of flu season, and a second wave that is longer and more severe.

This seems to imply that the same factors that are driving the seasonality of Influenza-Like Illnesses are causing COVID-19 seasonality as well, such as solar radiation (UV), temperature, relative humidity, and subsequently seasonal allergens and allergies.

## Highlights

• Time-series of COVID-19 and Influenza-Like illnesses have highly similar seasonal patterns in the Netherlands.
• COVID-19 satisfies the seasonal criteria of earlier flu-like pandemics.
• The implication is that the seasonal factors driving flu season, are also responsible for COVID-19 seasonality.
• Determined seasonality by applying comparative time-series analysis and a standardized logarithmic infection scale.

## 1 Introduction

During the first wave of COVID-19 it was hypothesized that COVID-19 is subject to multi-wave seasonality [1,2], comparable to other respiratory viral infections and pandemics since time immemorial [3,4]. It is observed that the COVID-19 community outbreaks have a similar pattern as other seasonal respiratory viruses [[5], [6], [7]]. Already during the first COVID-19 cycle the data suggested seasonality, comparable to the seasonality of Influenza-Like Illnesses (ILI), although the time-series were typically too short for definitive conclusions [8]. Currently, we are one year into the COVID-19 pandemic, and we can witness in the temperate climate zone in the Northern Hemisphere, a second wave that appears to rise and peak during the boundaries of a typical flu season, as the first cycle before.

Until now, it is not yet confirmed that COVID-19 behaves as seasonal as ILI. Therefore, we aim to test our hypothesis that COVID-19 has a similar seasonal pattern as ILI in a country in the temperate climate zone as the Netherlands. To test our hypothesis, we performed time-series analysis to compare the COVID-19 cycles with the multi-wave seasonality patterns of flu-like illnesses. In addition, we analyzed to what degree the COVID-19 pandemic fulfills the qualitative characteristics of earlier flu-like pandemics and seasonality as mentioned by Fox et al. Particularly, a short first wave at the tail end of a flu season, and a more severe second wave during the following flu season. We further expect peaks to occur within the seasonal boundaries between week 33 (± 2 weeks) and week 10 (± 5 weeks), and the nadir in the opposing period which coincides with the allergy season [8,9].

The main objective of this study is to provide a predictive model for subsequent COVID-19 seasonal cycles.

## 2 Methods

### 2.1 Data

#### 2.1.1 Incidence of influenza-like illnesses

We used data from the Dutch State Institute for Public Health (RIVM) gathered by the Dutch institute for research of the health care (Nivel) about weekly flu-like incidence (WHO code “ILI” - Influenza-Like Illnesses). ILI is defined by the WHO as a combination of a measured fever of ≥38 °C, and a cough, with an onset within the last 10 days. The Dutch ILI reports are gathered from primary medical care. Primary medical care is the first-line healthcare provided by general practitioners to their registered patients as typical in the Netherlands, with its current population of 17.4 million. The positive ILI results assessed via general practitioners are confirmed by a positive RIVM laboratory test for ILI. The ILI test covers influenza A and B strains, but also other viruses such as rhino viruses, enteroviruses, pseudo-influenza viruses, seasonal corona viruses [10].

**Fig. 1:**
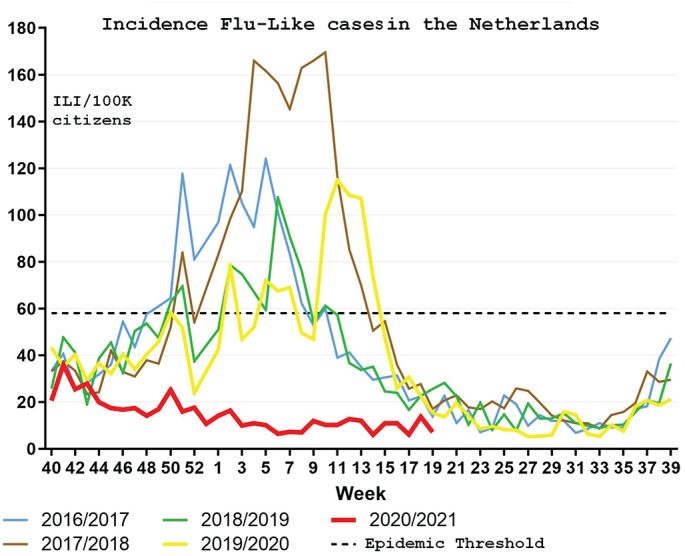
Overview of Influenza Like Illnesses (ILI) in the Netherlands from 2016 till 2021 based on NIVEL data. Whereby both the data gathering methods and incidence during the pandemic are heavily influenced because of lockdowns affecting access to primary care, standard referral of patients with ILI symptoms to COVID-19 test stations, and increased flu vaccination.

The flu-like incidence metric is a weekly average based on a representative group of 40 primary care units. It is calculated using the number of influenza-like reports per primary care unit divided by the number of patients registered at that unit. This is then averaged for all primary care units in the Netherlands, extrapolated to the entire population, and reported as the ‘ILI incidence per 100,000 citizens in the Netherlands’. The datasets run from week 1 of 2016 up to week 52 of 2019 to preclude the COVID-19 pandemic and avoid the impact of lockdowns and other measures on ILI incidence in the 2020/2021 season (see Fig. 1). Furthermore, it helps avoid collinearity caused by the inclusion of severe acute respiratory syndrome coronavirus 2 (SARS-CoV-2) in the metric from 2020 onward, and unreliability caused by referral of patients with ILI symptoms to COVID-19 test stations in stead. For earlier years, before 2016, we do not have complete ILI data sets at our disposal. We used these data to calculate the average ILI incidence per week (*n* = 52) as our baseline times series.

#### 2.1.2 COVID-19 incidence

To calculate the COVID-19 incidence, we used the RIVM data set which reports the daily COVID-19 incidence per municipality [11]. The incidence per municipality is based on positive COVID-19 tests that are reported via the local municipality health services (Gemeentelijke Gezondheidsdienst; GGD), that are under the control of the RIVM. We aggregated the crude number into a weekly COVID-19 incidence for the Netherlands per 100,000 citizens, to create a metric on the same scale as the standard ILI metric. We calculated the values from week 13, 2020, the peak of the short first COVID-19 cycle in the Netherlands, till week 5, 2021 (*n* = 45), before COVID-19 vaccination is scaled up. In week 5, still less than 2% of the Dutch population is vaccinated. We assume that the cycles themselves are sufficiently representative for time-series analysis, even though the COVID-19 incidence during the first cycle is most likely underestimated compared to incidence during the second cycle, due to test bias. With test bias, we mean that both the method of testing and the test capacity, altered during the development of the COVID-19 pandemic. Especially, at the start of the pandemic, there was a shortage of test capacity in the Netherlands. The capacity shortage had considerably improved since the end of March, i.e., week 13 of 2020, but only in the first months of 2021 reached sufficient levels.

Therefore, as a sensitivity analysis, we used a second dataset from RIVM, which is based on data from the Dutch national intensive care evaluation foundation [12]. Based on hospital admissions and subsequent serological tests per age group, the RIVM estimated the COVID-19 incidence in the Netherlands. The following assumptions are used: the incubation time of SARS-CoV-2 is between 4 and 10 days and a further delay of 7 days between first COVID-19 symptoms and hospital admission. This so-called “prevalence” dataset provides the average COVID-19 incidence per day with a 95% confidence interval (95% CI). Again, we calculated the average weekly COVID-19 incidence for the Netherlands per 100,000 citizens, to create a metric on the same scale as the standard ILI metric.

### 2.2 Statistical analysis

Variables are presented with their means (M) and standard deviations (SD). We calculated correlation coefficients to test the hypotheses and to assess the strength and direction of relationships.

Because the time-series were nonlinear and somewhat skewed, we used the log_10_ transformation before applying linear regression and calculating correlation coefficients, which requires a normal distribution. After the log_10_ transformation, we multiplied the data with factor 2 to create an intuitive scale from 0 to 10 for ILI and COVID-19, which is comparable to the 1 to 12 logarithmic Richter scale for earthquakes [13]. The logarithmic scale we elaborated is rational and plots exponential characteristics on a linear infections scale [14]. We added descriptive labels to each scale as an aid for qualitative interpretation in intuitive, layman terms (see Table 1). Other advantages are that it makes a comparison between different epidemics or pandemics, and thus external validation, easier. Finally, it enlarges the critical early stages of an epidemic, and it reduces the extreme peaks and resulting test bias because of test capacity overloads.

**Table 1:** Logarithmic infection scale.

2*LOG_**10**_**(Incidence/100** **K)**	**Incidence (/100** **K citizens)**	**Incidence (% of population infected)**	**Description**
0	1	0.001%	Isolated incidents
1	3	0.003%	Mild outbreak
2	10	0.01%	Moderate outbreak
3	30	0.03%	Severe outbreak
3.5	58	0.06%	Epidemic threshold (example)
4	100	0.1%	Mild Epidemic
5	300	0.3%	Moderate epidemic
6	1000	1%	Severe epidemic
7	3000	3%	Very severe epidemic
8	10,000	10%	Public health catastrophe
9	30,000	30%	Severe public health catastrophe
10	100,000	100%	Total public health catastrophe

Table 1: Logarithmic scale (1 to 10) of Influenza-Like Illnesses or COVID-19 (or other) incidence because of the exponential nature of epidemics, with proposed qualitative descriptions for convenience.

Linear regression (F-test) on the ILI and COVID-19 time-series is performed as a sensitivity analysis and used *descriptively* to determine the strength of the relationship between the COVID-19 and ILI time-series. More in detail to determine the equation using estimates and intercept values, probability, significance level, F-value, and the Multiple R squared correlation to understand the predictive power of the respective relation. Standard deviations and errors and degrees of freedom (DF) were used as input for calculating the 95% probability interval.

We have reported the results in APA style, adapted to journal requirements.

Correlations are calculated manually in Excel, and for linear regression Graphpad 2021 is used (which we benchmarked on R version 3.5).

## 3 Results

### 3.1 Data analysis

The means and standard deviations of the dataset are summarized in Table 2.

**Table 2:** overview means (M) and standard deviations (SDs).

			2*Log_**10**_**transformed**	
**Variable**	**Mean**	**SD**	**Mean**	**SD**
Average incidence ILI/100 K 2016 till 2019	46	36	3.07	0.69
COVID-19 incidence/100 K	126	141	3.34	1.43
COVID-19 incidence/100 K estimate based on hospitalizations (average)	415	337	4.69	1.17
COVID-19 incidence/100 K estimate based on hospitalizations (lower 95% CI)	300	247	4.38	1.22
COVID-19 incidence/100 K estimate based on hospitalizations (upper 95% CI)	531	426	4.93	1.14

Table 2: Overview of means (M) and standard deviations (SD) per variable in the ILI (Influenza-Like Illnesses) and COVID-19 datasets, including 2*Log_10_ transformed data.

**Fig. 2:**
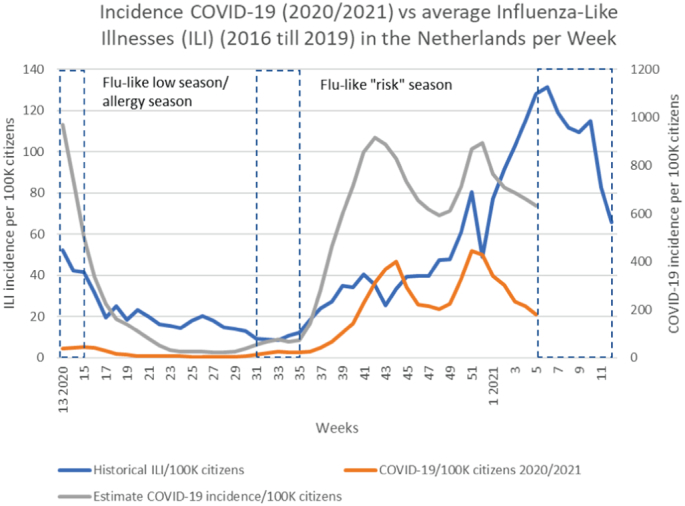
Historical ILI (Influenza-Like Illnesses) incidence (2016 till 2019) versus COVID-19 incidence per 100 K citizens during the 2020/2021 season. Peaks are all during flu season, and lows during the opposite season. The shaded periods are the typical period in which seasonal switching occurs.

Fig. 2 shows a short first COVID-19 wave at the tail end of the 2019/2020 flu season, and a more severe second wave during the 2020/2021 flu season regarding the estimated total incidence. The peaks are all within the seasonal boundaries between week 33 (± 2 weeks) and week 10 (± 5 weeks), and the nadirs in the opposing period. The hospitalizations-based estimates for COVID-19 incidence provide likely a more realistic picture of especially, the first wave, given test bias. However, on a logarithmic scale, the first COVID-19 wave appears visually more comparable in both time-series. Beyond the scope of our time series, the second wave has a third, somewhat lower peak, which ends, despite a relatively cold April month, in week 15 of 2021. From week 15 onward, the COVID-19 prevalence estimates and also the reproduction number (R_t_) is structurally below 1 since then. However, the GGD COVID-19 incidence data, shows a last peak one week later, but again suffered from a change in methodology as also positive results of commercial self-test kits were included during this third peak.

**Fig. 3:**
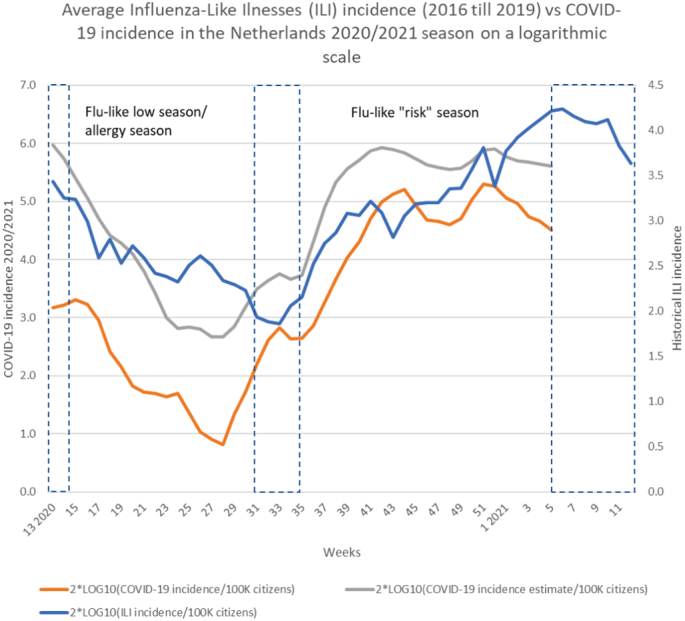
Historical ILI (Influenza-Like Illnesses) incidence (2016 till 2019) versus COVID-19 incidence per 100 K citizens (2020/2021) on the 1 to 10 logarithmic scale. This figure visualizes the similarity of the COVID-19 time-series based on hospitalizations with the historic ILI time-series. The shaded areas are the typical seasonal switching periods.

On our logarithmic infection scale (Fig. 3), the estimated COVID-19 incidence tops around 6 (severe epidemic level), and the nadir bottoms out around 3 (severe outbreak level). Interestingly, on this scale, it becomes visible that COVID-19 incidence starts to rise slightly earlier than what is usual for ILI (week 33 ± 2 weeks).

### 3.2 Statistical outcomes

**Fig. 4:**
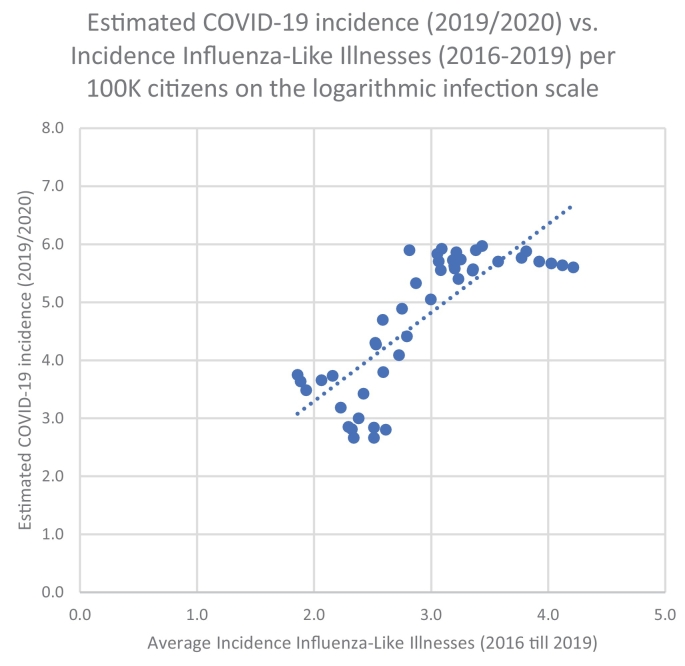
Scatter diagram showing the relation between the estimated COVID-19 incidence/100 K citizens and the seasonal, average incidence of Influenza-Like Illnesses (ILI)/100 K citizens in the Netherlands of the preceding 4 years.

The COVID-19 time-series strongly and highly significantly correlates with the ILI time-series *r*(45) = 0.75 (*p* < 0.00001). The hospitalizations-based, COVID-19 time-series, providing estimates that control for test bias, correlated even somewhat stronger, (*r*(45) = 0.798, *p* < 0.00001), and as significantly (Fig. 4). The correlations (95% CI) of the estimated COVID-19 incidence time-series are almost equal, respectively *r*(45) = 0.788, p < 0.00001 and *r*(45) = 0.803, *p* < 0.00001. Therefore, we conclude that the COVID-19 time-series have a similar wave pattern to the ILI time-series, which have long been established as being seasonal. Furthermore, the COVID-19 peaks, similar to ILI peaks, all occur during flu season, i.e., between week 33 (± 2 weeks) and week 10 (± 5 weeks). In addition, the COVID-19 nadirs, similar to ILI nadirs, occur all in the opposing allergy season.

As a second sensitivity analysis, we performed univariate regression analyses between both the COVID-19 time-series, and the average ILI time-series. The outcomes were again highly significant: respectively *F*(1, 43) = 61.45, *p* < 0.0001, and *F*(1, 43) = 81.18, p < 0.0001 and the correlations (*r*^*2*^) are moderate to strong (see Table 3).

**Table 3:** Outcomes linear regression.

2*LOG_10_(ILI/100 K)	Estimate	CI 95%	Intercept	R^2^	F-stat on DF	P<
2*LOG_10_(COVID-19 incidence/100 K)	1.81	1.34 to 2.28	1.95	0.59	61.45 (1, 43)	0.0001
2*LOG_10_(COVID-19 incidence/100 K) estimate based on hospitalizations	1.54	1.20 to 1.89	0.19	0.65	81.18 (1, 43)	0.0001

Table 3: Univariate regression analyses of the average Influenza-Like Illnesses (ILI) incidence/100 K citizens on both time-series for COVID-19 incidence/100 K citizens, whereby each time-series is 2*LOG_10_ transformed to compensate for non-linearity, and thus plotted on a 1 to 10 rational scale.

## 4 Discussion

Given the strong, and highly significant, correlations between the ILI and COVID-19 time-series, we conclude that COVID-19 incidence follows a seasonal pattern that is similar to ILI incidence in a country in the temperate climate zone, such as the Netherlands. Moreover, the COVID-19 peaks are all during flu season, and lows are all in the opposing period as expected. Furthermore, the COVID-19 time-series is in accordance with the two characteristics of earlier pandemics [4], namely a short first wave at the tail-end of a flu season, and a longer and more intense second wave during the subsequent flu season. This implies that the subsequent endings and starts of each following wave are more or less predictable. If the history of pandemics is followed, the third COVID-19 wave would be less severe than the second one. Though nowadays COVID-19 vaccination will be a more important factor in determining the amplitude of the subsequent waves. On the other hand, if the protective immunity is short-lasting as is typically the case with infections with the common coronavirus, we might still be confronted with resurgences of COVID-19 [15]. Nevertheless, it is likely that such new waves, would they occur, are less intense, given longer lasting B-cell and T-cell memory of people that have been infected or are vaccinated already.

Interestingly, all over Europe, the COVID-19 cycles were all more or less in sync with the Dutch COVID-19 cycle [16], and thus ILI seasonality, independent of the start of the first cycle, the severity of lockdown measures taken, and given that herd immunity is not yet reached. More in detail, the first short-lived COVID-19 cycle in Europe (conforming to WHO's definition of Europe), declines sharply during April 2020. The second cycle resurges from August, and peaks between the end of October and beginning of April, and declines sharply during the rest of April and May 2021. Also, the average Dutch flu season has the same pattern as the average European flu season [17].

The seasonality pattern of COVID-19 appears to be influenced though not caused by social distancing and lockdown measures as these measures were mainly anti-cyclical and following the trend. They were increasingly applied to flatten the curve after COVID-19 incidence increases, gradually lifted after the sharper than expected COVID-19 downcycles in Spring and Summer, and only re-applied after the second wave seriously kicked in, during Autumn and Winter. It is beyond our research to quantify the considerable impact of lockdown and social distancing measures, although it might explain that COVID-19 incidence on the logarithmic scale (see Fig. 3) starts to rise slightly earlier than what is usual for ILI (week 33 ± 2 weeks) as social distancing and lockdown measures were increasingly relaxed and ignored in this period. Given that we did not have comparable, historical ILI datasets available from areas on the southern hemisphere, in particular areas in Argentina and Chile, it is not meaningful within the scope of our research to compare these areas with the Netherlands. Moreover, in case of Australia and New Zealand, the strict lockdown measures succeeded in all but eradicate COVID-19, making a meaningful seasonal comparison nearly impossible.

What environmental factors have caused COVID-19 seasonality? We have analyzed before that the likely inhibiting factor causing ILI seasonality, before or during COVID-19, are seasonal allergens (i.e, pollens) and seasonal allergies [8,9], given that meteorological factors alone are not sufficient to explain the seasonality of ILI [18] or COVID-19 [19]. We used data from a pollen station in Helmond, the Netherlands (latitude 51.48167, longitude 5.66111). Spring in the Netherlands coincides with peaks in seasonal allergies. Pollens and allergy season typically ends around the beginning of August, and thus coincides with the typical flu-like low season period. On the other hand, we identified solar radiation (UV) as an ILI/COVID-19 co-inhibitor, and it is well-established that dry, warm, and sunny weather stimulates the maturation and dispersion of pollens.

The inverse seasonality of seasonal allergens and ILI including COVID-19 is independently confirmed by a recent Chicago (latitude 41.85003, longitude −87.65005) study that covered not only pollens but also mold spores [20]. These findings seemingly contradict an early international study of pollen and COVID-19 [21], which suffers from the same issues as many other early environmental COVID-19 studies: both sub-seasonal bias and COVID-19 test bias. Sub-seasonal bias, means here that a too limited time-series sample is used, namely from January till the beginning of April 2020, which coincides with only a small part of the early upswing in COVID-19 incidence in most countries, and mainly the upswing of allergens in the northern hemisphere. Additionally, conflicting local outcomes seem to have been selectively removed from the study. And, finally, in this short time window used it might have been better to correlate with a sound estimate of the reproduction number (R_t_), correcting for test bias, than only the raw incidence figures, to be able to discern deceleration/acceleration effects during the upswing.

We think that studies that are more geographically focused, and work with longer time-series, are in the current phase more meaningful for analyzing the pollen effect on COVID-19 incidence while controlling for deviations in other local circumstances. Therefore, we would expect that, ceteris paribus, the results of our COVID-19 and ILI pattern comparison in this study, implies that a 12-month time-series analysis of the reproduction number (R_t_) of COVID-19 and pollen concentrations would falsify the outcomes of this international study, at least for the Netherlands.

Upon the observation that allergic diseases are associated with lower rates of COVID-19 hospitalizations [26, 27], several pathophysiological explanations are provided: reduced expression of membrane-bound angiotensin-converting enzyme 2 (ACE-2) [28, 29] and. and Toll-Like Receptor 4 (TLR4) [20], the higher eosinophil count in patients with allergic diseases [30, 31],

reduced chance on a cytokine storm and hyper-inflammation [32], and, allergen proteins directing T cell-mediated heterologous immune responses [33].

#### 4.1.1 Methodological concerns

Test bias, especially for new viruses such as COVID-19, is a major methodological challenge. The approach to use more reliable metrics like the number of hospitalizations to generate an alternative incidence metric appears to be a good method to control for test bias. We could observe that the test bias slightly reduced the strength of correlations, which in our case did not affect the conclusions. During 2020–2021, the test capacity was scaled up significantly and sufficiently, which reduced the test bias concern over time.

Another sound approach used seems to be excess mortality estimates. In this study, we decided not to use the latter, as there are other known factors than seasonal viruses that cause excess mortality, such as the heatwave during the Summer of 2020. Thus, using excess mortality would just introduce a measurement validity concern.

Regarding the ILI data set, it would be better to have access to historical incidence data from before 2016, which would improve the calculation of the average ILI cycle, but also improves empirical insight in the historical variances from the means.

The end of the second COVID-19 season, beyond the scope of our dataset, around week 152,021, is not only influenced by seasonal factors, but also by the increased pace of vaccination, and the relaxation of lock-down measures. It will require additional data sets and multivariate variance analysis to try to determine the weight of each such factor, and their interactions.

## 5 Conclusion

The COVID-19 pandemic in the Netherlands is till now as seasonal as flu-like illnesses given the highly significant and strong correlations between both time-series. But, also given that COVID-19 waves till now rise between the temporal boundaries (week 10 ± 5 weeks and week 33 ± 2 weeks) of the typical flu-like season in The Netherlands, and go down in the opposing periods. Beyond `the scope of our selected time-series, it appears that the relatively cold April month of 2021 has lengthened the second wave slightly beyond the boundaries of our empirical model. Redefining the transition period from flu season to allergy season as week 11 (± 5 weeks) provides a slightly better fit with the available data.

Further, the COVID-19 pandemic satisfies the qualitative criteria of earlier respiratory pandemics since 1889: the first wave is short-lived at the tail-end of flu season, the second wave is longer and more severe, peaks fall within the boundaries of flu-like season, and the lows are during the boundaries of the opposing season.

This logically seems to imply that, ceteris paribus, the same factors that are driving the seasonality of Influenza-Like Illnesses are causing COVID-19 seasonality, such as solar radiation (UV), temperature, relative humidity, and subsequently seasonal allergens and allergies.

## Funding

This research did not receive any specific grant from funding agencies in the public, commercial, or not-for-profit sectors.

## Data statement

The links to the COVID-19 datasets are provided in the reference list and a reference to the source of Influenza-Like Illnesses. Upon request, the data used for this manuscript is available for inspection, but for other purposes we kindly refer to the respective copyright-holder(s).

## Declarations of Competing Interest

The authors declare that they have no known competing financial interests or personal relationships that could have appeared to influence the work reported in this paper.
